# Molecular transmission network and drug resistance analysis of 404 HIV-1 cases infected through commercial heterosexual contact (2020–2024)

**DOI:** 10.3389/fpubh.2026.1826363

**Published:** 2026-04-29

**Authors:** Lian Ma, Hui Liu, Xingjing Gao, Fan Zhang

**Affiliations:** 1 College of Public Health, Chongqing Medical University, Chongqing, China; 2 Yubei District Center for Disease Control and Prevention, Chongqing, China; 3 Research Center for Medicine and Social Development, Chongqing Medical University, Chongqing, China

**Keywords:** commercial heterosexual contact, genotype, HIV-1, molecular transmission network, pretreatment drug resistance

## Abstract

**Background:**

HIV/AIDS remains a significant public health challenge. This study aimed to investigate the molecular transmission network and drug resistance characteristics among HIV-1 infected individuals through commercial heterosexual contact in Yubei District which located in a southwest city in China, thereby assessing HIV transmission risks in the region.

**Methods:**

Blood samples were collected from newly diagnosed HIV-1 patients in Yubei District in Chongqing city between 2020 and 2024, all of whom acquired the infection through commercial heterosexual contact. The HIV-1 pol region was amplified and sequenced to construct a phylogenetic tree. Drug resistance mutation sites were obtained by comparing with the Stanford Drug Resistance database. A molecular transmission network was constructed using a genetic distance threshold of 1.3%. Univariate and multivariate logistic regression analyses were used to determine potential factors influencing network entry and the formation of large clusters.

**Results:**

Among the 404 sequences, CRF07_BC was the predominant genotype (55.9%). The pretreatment drug resistance rate was 4.70% for all cases of commercial heterosexual exposure. Molecular network analysis revealed that 223 sequences (with an entry rate of 55.2%) formed 39 clusters, including 6 large clusters with more than 10 nodes. Multivariate logistic regression models identified age ≥ 60 years, household registration in Yubei District, and CRF85_BC genotype as risk factors for network inclusion and large cluster formation. Additionally, the study identified a transmission cluster with significant geographic aggregation, featuring one female sex worker who posed a high transmission risk.

**Conclusion:**

HIV-1 infections transmitted through commercial heterosexual contact exhibit pronounced geographic clustering, with over half of the cases belonging to the CRF07_BC genotype. Although the pretreatment drug resistance prevalence remains low, establishing a sustained resistance surveillance system remains essential. Targeted management and precise interventions are required for identified high-risk transmission networks to effectively curb further spread of HIV.

## 1 Introduction

2025 Global AIDS Update—AIDS, Crisis and the Power to Transform, released by the Joint United Nations Programme on HIV and AIDS (UNAIDS) indicated that by the end of 2024, there had been approximately 40.8 million People Living With HIV (PLWHA) globally, with around 1.3 million new infections. Although this represents a 38% decrease compared to 2010, 9.2 million people still lack access to treatment (1, 2). By mid-2024, China had reported 1.329 million PLWHA (3). Currently, China has yet to fully achieve the UNAIDS “95 - 95 - 95” targets for diagnosis, treatment, and viral suppression rates among infected individuals (4, 5). Chongqing, one of the four directly administered municipalities in China, serves as a major transportation hub and economic center with dense populations and high mobility. Consequently, it had rapidly emerged as one of the regions with the most severe HIV-1 epidemics in the country (6). Local AIDS reporting system data indicate that Yubei District ranks second in Chongiqng for epidemic severity. And there were 95% of PLWHA infected by sexual contact.

Commercial Heterosexual Contact (CHC) is a major form of heterosexual transmission and the main route promoting the spread of HIV (7). The CHC transmission pattern is particularly prominent among female sex workers (FSWs) and older adults. The sexual networks of these populations frequently overlap with the general public, thereby acting as key transmission bridges for HIV (8, 9). It has been reported (10) that the proportion of PLWHA infected through CHC in China had significantly increased among >65 years old group. Local data showed that older adults account for 16.4% of the total population in Yubei District. Therefore, a thorough understanding of the distribution status of CHC transmission among PLWHA in this area is of great significance for formulating targeted prevention and control strategies.

Since the national rollout of free antiretroviral treatment (ART) for HIV, the prevalence of drug resistance mutations (DRMs) rose significantly nationwide (11, 12). Pretreatment drug resistance (PDR) refers to drug resistance detected in individuals who have never received ART or have prior antiretroviral drug exposure (13). In 2020, the PDR prevalence among HIV positive individuals in Chongqing reached 10.54%, exceeding the national average (7.4%), indicating moderate level of drug resistance (5, 14, 15). Furthermore, the prevalence of PDR for non-nucleoside reverse transcriptase inhibitors (NNRTIs) is concerning, as NNRTIs are a key component of the current current first-line regimen promoted by the Chinese government. Regular PDR monitoring enables timely assessment of the regimen’s effectiveness, which facilitates further adjustments (14). Molecular transmission network analysis utilizes genetic similarity of viral sequences to infer transmission links among individuals. Since the release of the “Technical Guidelines for HIV Transmission Network Surveillance and Intervention” (16) in 2019, molecular transmission network surveillance technology has been progressively implemented nationwide. Recently, the National Center for AIDS/STD Control and Prevention updated these guidelines, underscoring China’s commitment to applying molecular transmission network technology for HIV prevention and control (17). This technology can be used to monitor HIV drug resistance, analyze the transmission chains and identify high-risk geographic areas (18).

Commercial heterosexual contact, due to its covert nature and high infection risk, has remained poorly understood in terms of its transmission patterns at the molecular epidemiological level. This study analyzed the CHC cases reported in Yubei District, Chongqing, between 2020 and 2024, using drug resistance and molecular transmission network analysis. This study aims to identify drug resistance sites, transmission hotspots and cluster characteristics. The findings offer direct evidence for risk assessment and targeted interventions, providing a scientific basis for optimizing prevention strategies.

## 2 Methods

### 2.1 Study population and sample collection

Newly reported HIV-1 infected individuals in Yubei District, Chongqing Municipality from 2020 to 2024 were selected as study subjects. The inclusion criteria were as follows: infection source exclusively from commercial heterosexual contact; age ≥ 18 years; initiating or restarting ART for the first time; and having signed an informed consent form. After sample coding, basic demographic information was collected, including gender, age, occupation, marital status, education, household registrations and number of commercial heterosexual partners. Finally, we collected 10 mL of blood from each participant, set aside 1 mL for CD4^+^ T-cell counting, and centrifuged the remainder; the resulting plasma was stored in a − 80 °C freezer. The flow cytometry was employed to determine CD4^+^ T cell counts.

### 2.2 Extraction, amplification, and sequencing

RNA extraction was performed using the QIAamP Viral RNA Mini Kit. Reverse transcription and first-round Polymerase Chain Reaction (PCR) were conducted on a partial fragment of the pol region (HXB2:2,147–3,462) using the TaKaRa One SteP RNA PCR Kit (AMV). Cycling conditions were 50 °C for 30 min; 94 °C for 5 min; 94 °C for 30s, 55 °C for 30s, 72 °C for 2.5 min, 30 cycles; followed with an extension at 72 °C for 10 min. A second-round PCR was subsequently performed using 2×Taq PCR Mix (TIANGEN, China). Cycling conditions were 94 °C for 5 min; 94 °C for 30s, 63 °C for 30s, 72 °C 2.5 min, 30 cycles; followed with an extension at 72 °C for 10 min. Successfully amplified products were sent to Beijing Biomad Gene Technology Co., Ltd. for purification and gene sequencing, which was performed on an ABI 3730xl automated DNA analyzer (Applied Biosystems, Foster City, CA, USA).

### 2.3 Genetic subtype identification

All obtained raw sequences were first quality-controlled using Sequencher 5.4.6 software (Gene Codes Corporation, Ann Arbor, MI, USA). Sequences with a minimum length of 1,000 bp or ambiguous base (N) content exceeding 5% were excluded from further analysis. The remaining high-quality sequences were assembled using Sequencher 5.4.6 software. Subsequently, all sequences were aligned automatically via the ClustalW tool in BioEdit 7.0.9 (19), followed by manual editing. Genotypic determination of sequences was performed using the online Context-based Modeling for Expeditious Typing (^1^COMET) (20). Sequences were defined as uncertain if they had a COMET confidence score < 70%, a “NA” support value, or an ambiguous subtype assignment. For these uncertain sequences, the HIV Basic Local Alignment Search Tool (^2^BLAST) (21) was used for final classification. Subsequently, a phylogenetic tree was generated using the Tamura-Nei 93 (TN93) model in MEGA 7.0 with the Neighbor-Joining method (22), referencing sequences from the ^3^HIV database. The reliability of the phylogenetic tree was assessed using the bootstrap test with 1,000 replicates. Bootstrap values greater than 70% were considered to indicate strong statistical support for the corresponding nodes. Finally, the phylogenetic tree was visualized using the ^4^ITOL (23).

### 2.4 Drug resistance mutation analysis

The Stanford Drug Resistance Database^5^ was used to assess the resistance of HIV patients to three classes of antiretroviral drugs: nucleoside reverse transcriptase inhibitors (NRTIs), non-nucleoside reverse transcriptase inhibitors (NNRTIs), and protease inhibitors (PIs). HIV-1 strains require mutations at more than one resistance site to be considered resistant (19).

### 2.5 Construction of molecular network

Target sequences were analyzed using HyPhy 2.2.4 software (24) and the TN93 model was selected to calculate the pairwise genetic distances. Pairwise genetic distance calculated using the TN93 model provides a fast computation that takes into account variations in base composition and the differential rates of the two types of transitions. A sensitivity analysis of genetic distance was conducted. We tested thresholds ranging from 0.1% to 2.0% at intervals of 0.1%. When the genetic distance was 1.3%, there are the maximum number of clusters while preventing the formation of overly large clusters. This threshold is in line with the previously reported optimal range of genetic distance (25). Visualization was achieved using Cytoscape 3.7.2 (26).

### 2.6 Statistical analysis

All statistical analyses were performed using SPSS software (version 25.0). Univariate and multivariate logistic regression analyses were used to identify the factors influencing sequence clustering. Variables with *p* < 0.05 in univariate logistic regression were included in multivariate regression analysis. Ultimately, a *p* value less than 0.05 was considered statistically significant.

## 3 Results

### 3.1 Sociodemographic characteristics

**Figure 1:**
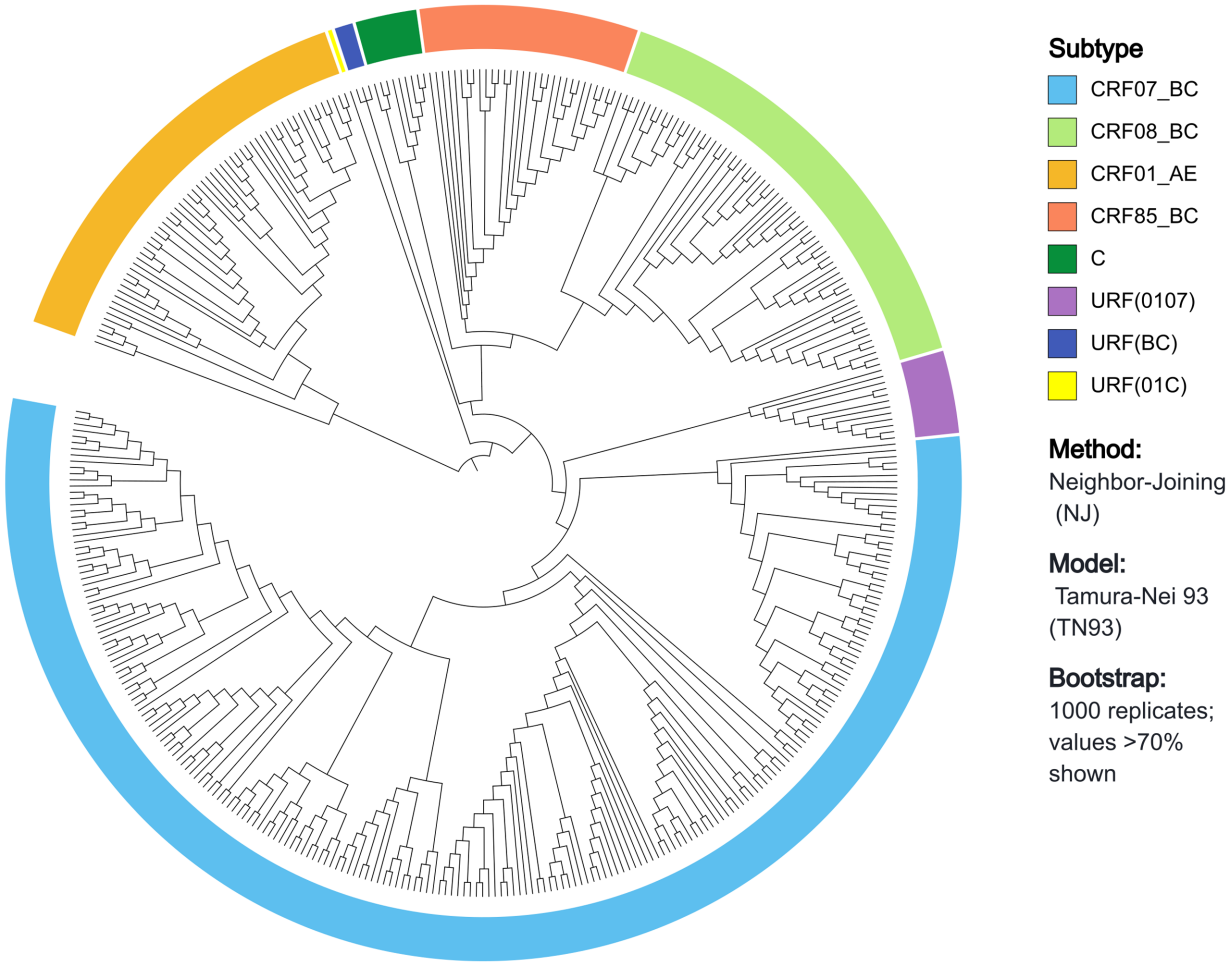
HIV-1 genetic subtype distribution in the phylogenetic tree.

A total of 461 samples were included in this study, and 404 pol region gene sequences were successfully obtained, with a success rate of 87.64%. The average age of these samples was (65.18 ± 13.1) years, with the majority being over 60 years old. Most of the participants were male (96.8%). Most participants’ registered residence was in Yubei District, Chongqing (60.9%). 42.6% of the participants were homemakers and unemployed. The marital status was married for 53.5% of the patients. The education level was elementary school or below for 52.0% of the participants. First CD4^+^ T cell counts ranged from 200 to 500 cells/μL in 46.8% of the patients (Table 1). The phylogenetic tree revealed prevalent strain subtypes including CRF07_BC (55.9%, 226/404), CRF08_BC (15.6%, 63/404), CRF01_AE (14.6%, 59/404), and CRF85_BC (7.6%, 31/404), followed by Subtype C and URFs. The three URFs were URF (0107), URF (BC), and URF (01C), collectively accounting for approximately 4% (Figure 1).

**Table 1:** Analysis of factors associated with clustering in the molecular network of individuals with HIV in Yubei District.

Variables	Subjects(%) *n* = 404	Clustering(%) *n* = 223	Univariate analysis		Multivariate analysis	
			*OR* (95%*CI*)	*p*	*OR* (95%*CI*)	*p*
Gender						
Male	391 (96.8)	218 (55.8)	1	0.124		
Female	13 (3.2)	5 (38.5)	0.496 (0.159–1.543)	0.226		
Age						
16 ~ 59	134 (33.2)	55 (41.0)	1	0.039	1	
≥60	270 (66.8)	168 (62.2)	2.366*** (1.550–3.612)	<0.001	2.025** (1.227–3.343)	0.006
Occupation						
Peasant	118 (29.2)	78 (66.1)	1	0.013	1	
Housework and Unemployment	172 (42.6)	91 (52.9)	0.576* (0.355–0.935)	0.026	0.710 (0.417–1.208)	0.207
Others	114 (28.2)	54 (47.4)	0.462** (0.272–0.784)	0.004	0.853 (0.457–1.596)	0.620
Marital status						
Single	70 (17.3)	41 (58.6)	1	0.44		
Divorced or widow	118 (29.2)	70 (59.3)	1.094 (0.601–1.991)	0.769		
Married	216 (53.5)	112 (51.6)	0.823 (0.478–1.417)	0.482		
Education						
Illiteracy	42 (10.4)	28 (66.7)	1	0.032	1	
Primary or below	210 (52.0)	72 (34.3)	0.707 (0.352–1.420)	0.33	0.945 (0.445–2.006)	0.882
Junior or above	152 (37.6)	123 (80.9)	0.450* (0.220–0.921)	0.029	0.764 (0.344–1.697)	0.509
Household registrations						
Yubei District	246 (60.9)	159 (64.6)	1	<0.001	1	
Other districts of Chongqing	125 (30.9)	52 (41.6)	0.390*** (0.251–0.606)	<0.001	0.407*** (0.250–0.664)	<0.001
Other provinces	33 (8.2)	12 (36.4)	0.313** (0.147–0.666)	0.003	0.368* (0.161–0.839)	0.017
Number of commercial heterosexual partners						
1 ~ 2	132 (32.7)	71 (53.8)	1	0.276		
3 ~ 5	177 (43.8)	104 (58.8)	1.253 (0.795–1.975)	0.331		
>5	95 (23.5)	48 (50.5)	0.841 (0.496–1.426)	0.521		
Genetic subtype						
CRF07_BC	226 (55.9)	129 (57.1)	1	0.002	1	
CRF08_BC	63 (15.6)	27 (42.9)	0.564* (0.321–0.991)	0.047	0.562 (0.309–1.023)	0.059
CRF01_AE	59 (14.6)	26 (44.1)	0.592 (0.333–1.056)	0.076	0.570 (0.306–1.061)	0.076
CRF85_BC	31 (7.7)	27 (87.1)	5.076** (1.719–14.985)	0.003	5.878** (1.922–17.978)	0.002
Others^a^	25 (6.2)	14 (56.0)	0.957 (0.416–1.200)	0.918	0.746 (0.306–1.822)	0.520
First CD4^+^ T cell count (cells/μL)						
<200	162 (40.1)	93 (57.4)	1	0.671		
200 ~ 500	189 (46.8)	99 (52.4)	0.855 (0.561–1.304)	0.467		
>500	53 (13.1)	31 (58.5)	1.072 (0.572–2.010)	0.828		

*p < 0.05, **p < 0.01, ***p < 0.001; ^a^Others include URF(0107), URF(01C) URF(BC), and Subtype C (*n* = 11, 1, 5, 8).

### 3.2 Analysis of drug resistance characteristics

**Figure 2:**
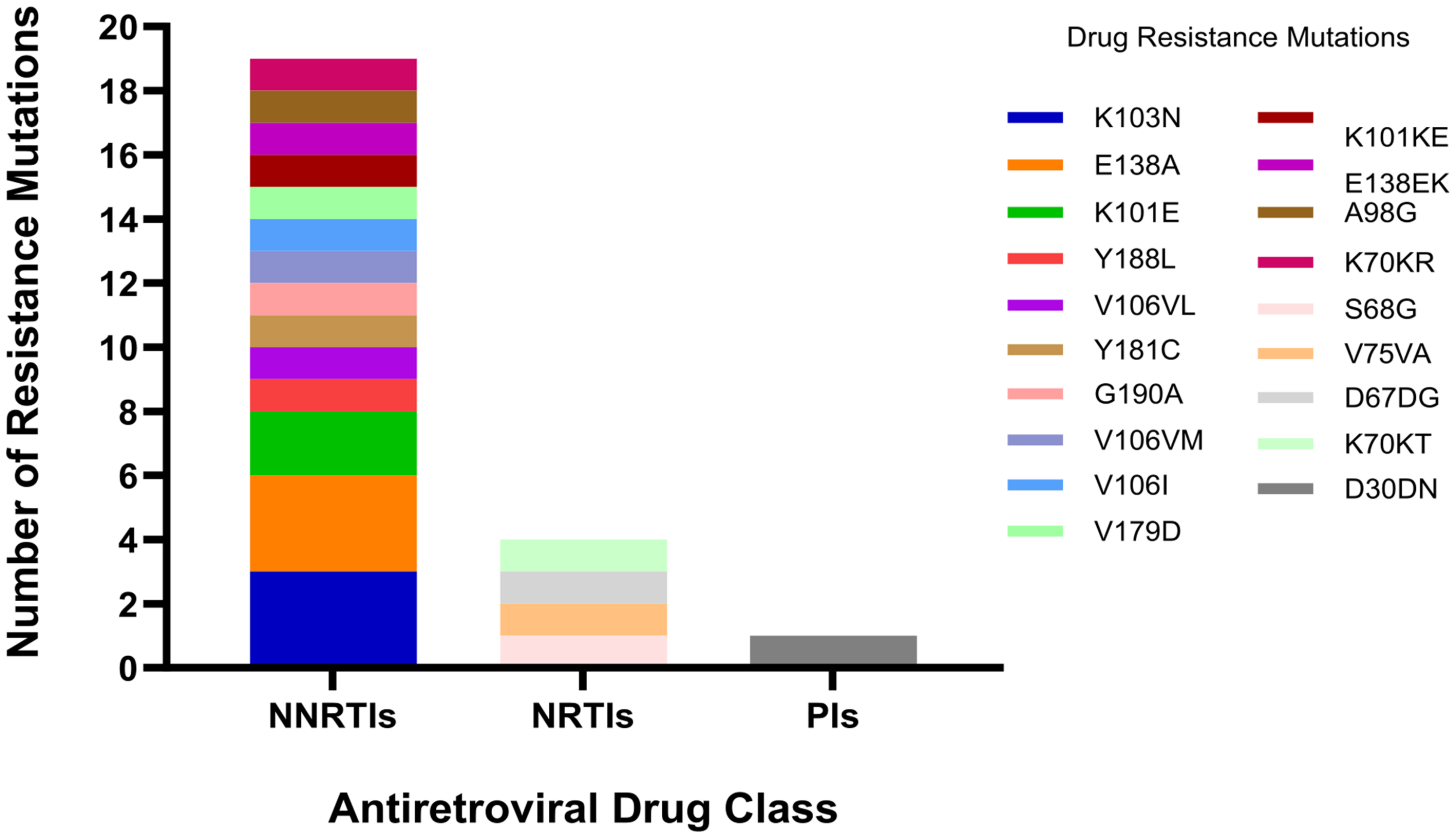
Distribution of HIV-1 pretreatment drug resistance mutations by antiretroviral drug class.

Among the 404 patients with CHC, 57 had DRMs. The primary DRM sites were V179D (3.96%, 16/404), V179E (1.49%, 6/404), and S68G (1.49%, 6/404). NNRTIs exhibited the highest number of mutation sites, including V179D (33.3%, 16/48), V179E (12.5%, 6/48), and V179VD (8.3%, 4/48). Additionally, 19 cases (4.70%, 19/404) were related to PDR. Among these, NNRTIs-associated PDR mutations had the highest proportion (3.47%, 14/404), followed by NRTIs (1.00%, 4/404) and PIs (0.25%, 1/404). Figure 2 showed the distribution of mutation sites related to different antiretroviral drugs.

### 3.3 Construction of molecule transmission networks

**Figure 3:**
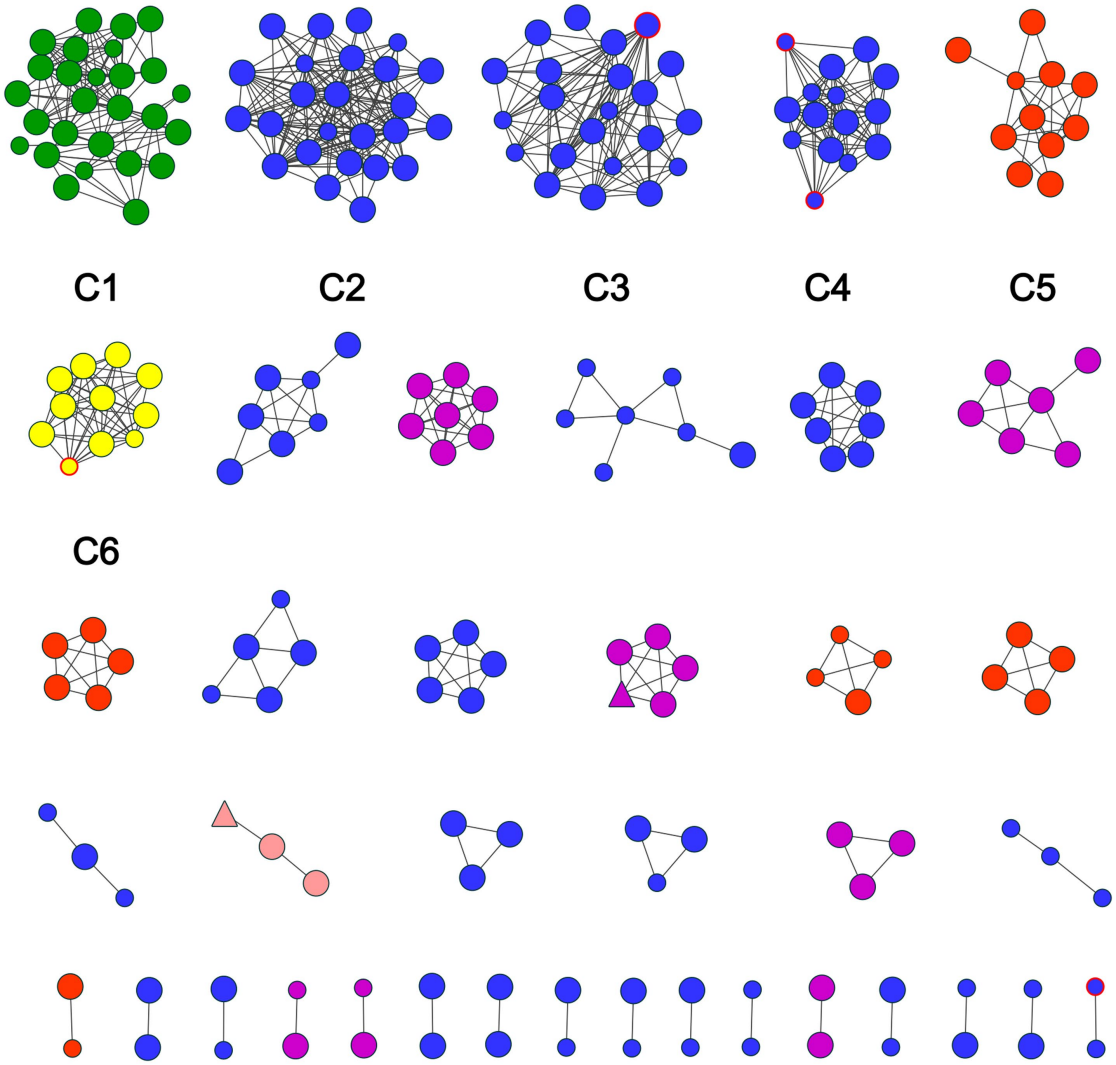
Molecular transmission network of sequences. Red borders indicate females. Smaller figures represent ages 16–59, while larger figures indicate ages >60. Triangles represent PDR mutations, while circles represent no PDR mutations. Different colors represent distinct genotypes: green indicates CRF85_BC, blue indicates CRF07_BC, purple indicates CRF08_BC, orange indicates CRF01_AE, pink indicates Subtype C, and yellow indicates URF (0107).

When the gene threshold was set at 1.3%, a total of 39 transmission clusters were identified, comprising 223 sequences (55.2%, 223/404) and 698 edges. The node counts within clusters ranged from 2 to 27. Among all transmission clusters, 16 clusters (41.0%) comprised two nodes, 17 clusters (43.6%) comprised three to seven nodes, and 6 clusters (15.4%) comprised ≥ 10 nodes. The largest transmission cluster (C1), comprised 27 nodes (showed in Figure 3). All nodes in the molecular transmission network had degrees ranging from 1 to 24, with nodes having ≥ 4 degree (designated as individuals with high transmission risk) accounting for 62.3% (139/223). Additionally, the study identified only two PLWHA with NNRTI drug resistance among all the network members.

### 3.4 Analysis of risk factors for transmission networks

Univariate and multivariate logistic regression analyses were conducted to examine the factors influencing clustering (Table 1). Multivariate logistic regression revealed that individuals ≥ 60 years were significantly more likely to cluster than those under 60 years (*OR* = 2.025, 95%*CI* = 1.227–3.343, *p* = 0.006). Patients registered in other districts of Chongqing and those registered in other provinces had lower clustering probabilities than those registered in Yubei District, with OR values of 0.407 (*p* < 0.001) and 0.368 (*p* = 0.017), respectively. Compared with individuals with the CRF07_BC genotype, those with CRF85_BC were more likely to cluster (*OR* = 5.878, 95%*CI* = 1.922–17.978, *p* = 0.002). Gender, occupation, marital status, education, number of commercial heterosexual partners, and CD4^+^ T cell count at first detection had no statistically significant effect on individual clustering.

### 3.5 Characteristics analysis of large transmission clusters

**Figure 4:**
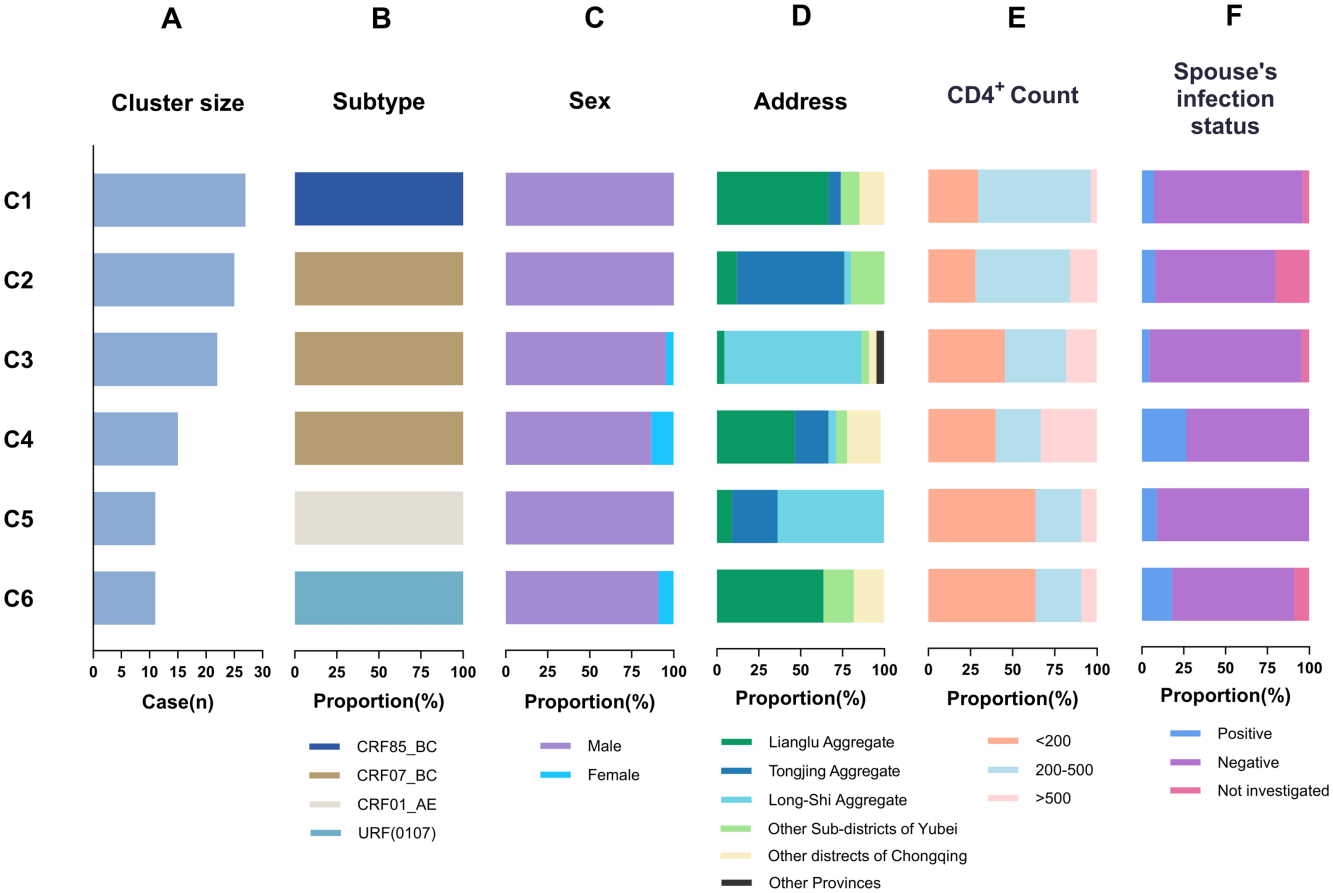
The core features of large molecular transmission clusters. Lianglu aggregate, combining the three sub-districts of Shuanglonghu, Huixing, and Shuangfengqiao; Tongjing aggregate, combining the two towns of Gulu and Dasheng; Long-Shi aggregate, combining the two towns of Longxing and Shichuan.

In this study, 6 large clusters (nodes ≥ 10) comprised 111 individuals, encompassing four genotypes: CRF85_BC, CRF07_BC, CRF01_AE, and URF (0107). No PDR distribution was observed within the large clusters. As shown in Table 2, individuals < 60 years old exhibited a lower risk of inclusion in the large clusters compared to those aged ≥ 60 years (*OR* = 1.925, 95%*CI* = 1.024–3.618, *p* = 0.042). Patients registered in other districts of Chongqing (*OR* = 0.372, *p* = 0.003) and other provinces (*OR* = 0.109, *p* = 0.006) had a lower risk of inclusion than local residents of Yubei District. Compared with CRF07_BC, CRF85_BC had a significantly higher likelihood of inclusion (*OR* = 30.568, 95%*CI* = 9.007–103.748, *p* < 0.001). Additionally, Figure 4 illustrated the distribution of core characteristics within large clusters. All 6 large clusters exhibited a higher proportion of males. The highest proportion of patients resided in the Yubei District (Lianglu Aggregate, Tongjing Aggregate, Long-Shi Aggregate, and other towns/sub-districts of Yubei District), accounting for 90.1% of the total. However, patients from other districts in Chongqing Municipality and other provinces were also included in some of the large clusters. Cases with positive partners were identified in 6 clusters. Furthermore, this study identified distinct geographic clustering within the key transmission clusters. Cluster C3 comprised 22 infected individuals (1 female and 22 males), predominantly concentrated in the Long-Shi Aggregate, with 7 cases residing in the same residential complex. No PDR mutations were detected in any case.

**Table 2:** Characteristics of the large molecular transmission clusters (*n* = 111).

Variables	Subjects(%) *n* = 404	Included in a large cluster (%, *n* > 10)	Univariate analysis		Multivariate analysis	
			*OR* (95%*CI*)	*p*	*OR* (95%*CI*)	*p*
Gender						
Male	391 (96.8)	107 (27.4)	1			
Female	13 (3.2)	4 (30.8)	1.180 (0.356–3.911)	0.787		
Age						
16 ~ 59	134 (33.2)	22 (16.4)	1		1	
≥60	270 (66.8)	89 (33.0)	2.503*** (1.484–4.222)	<0.001	1.925* (1.024–3.618)	0.042
Occupation						
Peasant	118 (29.2)	43 (36.4)	1	0.015	1	0.178
Housework and Unemployment	172 (42.6)	46 (26.7)	0.637 (0.384–1.055)	0.080	0.605 (0.334–1.095)	0.097
Others	114 (28.2)	22 (19.3)	0.417** (0.229–0.758)	0.004	0.553 (0.259–1.181)	0.126
Marital status						
Single	70 (17.3)	18 (25.7)	1	0.394		
Divorced or widow	118 (29.2)	36 (30.5)	1.372 (0.709–2.657)	0.348		
Married	216 (53.5)	55 (25.5)	0.987 (0.532–1.830)	0.967		
Education						
Illiteracy	42 (10.4)	14 (33.3)	1	0.124		
Primary or below	210 (52.0)	64 (30.5)	0.877 (0.433–1.775)	0.715		
Junior or above	152 (37.6)	33 (21.7)	0.555 (0.262–1.172)	0.123		
Household registrations						
Yubei district	246 (60.9)	85 (34.6)	1	<0.001	1	<0.001
Other districts of Chongqing	125 (30.9)	23 (18.4)	0.427** (0.253–0.721)	0.001	0.372** (0.192–0.719)	0.003
Other provinces	33 (8.2)	3 (9.1)	0.189** (0.056–0.639)	0.007	0.109** (0.022–0.530)	0.006
Number of commercial heterosexual partners						
1 ~ 2	132 (32.7)	44 (33.3)	1	0.038	1	0.191
3 ~ 5	177 (43.8)	50 (28.2)	0.787 (0.483–1.283)	0.337	0.853 (0.480–1.517)	0.589
>5	95 (23.5)	17 (17.9)	0.436* (0.230–0.824)	0.011	0.507 (0.242–1.062)	0.072
Genetic subtype						
CRF07_BC	226 (55.9)	62 (27.4)	1	<0.001	1	<0.001
CRF01_AE	59 (14.6)	11 (18.6)	0.606 (0.296–1.242)	0.171	0.576 (0.268–1.235)	0.156
CRF85_BC	31 (7.7)	27 (87.1)	17.855*** (6.003–53.103)	<0.001	30.568*** (9.007–103.748)	<0.001
URF (0107)	11 (2.7)	11 (100)	0.378** (0.188–0.758)	0.006	0.363** (0.176–0.748)	0.006
Others	77 (19.1)	0 (0)	–	–	–	–
First CD4 + T cell count (cells/μL)						
<200	162 (40.1)	45 (27.8)	1	0.860		
200 ~ 500	189 (46.8)	50 (26.5)	0.935 (0.583–1.499)	0.781		
>500	53 (13.1)	16 (30.2)	1.124 (0.570–2.219)	0.735		

*p < 0.05, **p < 0.01, ***p < 0.001.

## 4 Discussion

This study found that the most prevalent genotype among HIV-1 cases infected via CHC was CRF07_BC, consistent with the dominant transmission patterns in Chongqing, Sichuan, and Guangzhou (27, 28). CRF07_BC originated from drug addicts in Yunnan Province (29, 30) and rapidly spread to other provinces (31), and its prevalence has surpassed that of CRF01_AE in recent years (32). A molecular surveillance report from eastern China indicates that CRF07_BC is the predominant genotype among patients with CHC within major molecular clusters (33), consistent with our findings. Additionally, CRF08_BC and CRF01_AE accounted for 15.5 and 14.6% of the cases in this study, respectively. CRF08_BC shared a common origin with CRF07_BC, whereas CRF01_AE was initially introduced into Yunnan Province by sex workers from Southeast Asia (34). The study identified eight genetic subtypes in patients with CHC, demonstrating genetic diversity. As a core transportation hub bordering high-prevalence provinces like Yunnan and Guizhou, Chongqing experiences frequent population mobility, which has intensified the risk of viral transmission and recombination between regions. Therefore, monitoring, case identification and intervention measures need to be developed for PLWHA to curb the emergence and spread of novel HIV-1 recombinants.

The PDR prevalence among patients with CHC (4.70%) was slightly lower than the national average for PLWHA (5.56%) (35). However, this cross-sectional comparison does not allow us to attribute to difference causally to specific prevention and control measures, as the observed difference may reflect secular trends in PDR prevalence, regional variation in HIV testing coverage, or differences in ART uptake and regimen composition. Future studies should incorporate longitudinal surveillance data and adjust for these confounders to better evaluate targeted prevention strategies in this region. NNRTIs accounted for the highest proportion of PDR mutations (3.27%), followed by NRTIs (1.00%) and PIs (0.25%). This distribution aligned with the drug resistance profiles observed in newly reported infections in certain provinces (36, 37), but differs from that in MSM populations (38, 39). Reported data indicate that the proportion of MSM patients with PI resistance is often higher than that of NRTI resistance (in contrast to our finding that NRTI mutations outnumbered PI mutations). It is possible that MSM in many regions undergo more frequent HIV testing and initiate treatment earlier. Some patients switch to enhanced PI-based regimens after first-line treatment failure (11). In this study, the primary NNRTI mutation sites were V179D, V179E, and V179VD. The prevalence of V179 mutations may be associated with the long-term use of EFV as a first-line backbone drug in China (40). Therefore, alongside high-coverage treatment strategies, a sustainable drug resistance monitoring system is imperative.

Molecular network analysis revealed that the entry rate of all sequences was 55.2%, which was higher than the 40.5% reported for Ningbo City at the same threshold (41). At a genetic distance of 1.3%, PLWHA infected with CHC exhibited clustering. Within these clusters, most individuals had ≥ 4 links (62.3%), indicating high transmission capacity. These findings suggest that prevention efforts focus on identifying and interrupting high-risk population and clustered networks. Age, registered residence, and genotype were independent factors influencing the clustering. Specifically, age ≥ 60 years and registered residence in Yubei District significantly increase the risk of network entry, which is consistent with the conclusions of previous studies (42, 43). Older individuals infected through CHC play a key role in HIV transmission. Older age is associated with a higher likelihood of being in a cluster (44). This cohort predominantly comprises older men with low mobility, poor HIV awareness and high rates of widowhood or divorce. These factors drive their reliance on commercial sex services (45). Older men’s commercial sexual activities predominantly occur in low-end venues, indicating that AIDS prevention departments should prioritize regular interventions in rental housing, small inns, massage parlors and other venues associated with clandestine prostitution, while strengthening condom promotion and usage. Regarding genotypes, CRF85_BC exhibited a significantly higher risk of entering the network than other subtypes. This subtype originated in Yunnan, was first detected in Sichuan where it formed local transmission chains, and is prevalent among heterosexual populations (46). A Sichuan (47) study reported commercial heterosexual contact as primary transmission route for CRF85_BC, consistent with our findings. Sichuan and Chongqing are geographically adjacent, posing a risk of cross-regional spread of infectious diseases. Enhanced cross-regional monitoring and management between the two areas are necessary. It is worth noting that in the multivariate model, the effects of variables, such as gender, occupation, marital status, education, number of commercial heterosexual partners, and CD4^+^ T cell count at first detection on clustering were not statistically significant. In epidemiological studies, statistical non-significance does not necessarily imply the absence of a biological association. For instance, the small number of female participants (*n* = 13) in this study might have resulted in insufficient statistical power to detect potential gender-specific effects. Similarly, variables such as the number of commercial heterosexual partners obtained through self-reporting may have had measurement errors, leading to distorted results. Future research should consider increasing the sample size, adopting more precise or objective measurement methods, or conducting longitudinal studies to further explore the potential roles of these factors. Additionally, only two cases of NNRTI DRMs were identified among all samples, indicating a low prevalence of DRMs in this CHC patient population.

Age, registered residence, and genotype were the influencing factors of large clusters (nodes ≥ 10). Although gender did not affect clustering, the proportion of females was significantly lower than that of males. This may be because when the transmission route was CHC, FSWs exhibit high mobility and concealment, making them difficult to identify and enroll in the network. Furthermore, in all 6 large clusters, male infected individuals with HIV-positive spouses were identified. Men infected with CHC had a high probability of transmitting HIV to their healthy partners (44). This underscored the necessity of advancing the implementation of couple HIV counseling and testing strategies (47). All clusters exhibited distinct geographic correlations. Cases in C1, C4, and C6 were mainly concentrated in the Lianglu Aggregate; C2 was mainly populated by residents from the Tongjing Aggregate; and C3 and C5 cases were clustered in the Long-Shi Aggregate. This suggests the need to collaborate with local police to crack down on prostitution and increase testing among high-risk populations to facilitate early detection. A small number of imported cases from other districts of Chongqing and other provinces were identified within 6 clusters. This suggests that health authorities need to establish regional case tracking and information-sharing platforms, and strengthen cross-regional joint prevention for more precise interventions (48, 49). Notably, within cluster C3 (corresponding to CRF07_BC), one female was linked to 21 older males. Seven individuals, including this woman, resided in the same residential compound. An interview with this woman revealed she had engaged in commercial sex with 50 male partners, confirming her status as a FSW. Furthermore, the degree centrality (cD) of this FSW within C3 was calculated as 0.71, indicating a high risk of propagation. Follow-up should continuously monitor and investigate this network to track associated individuals and interrupt potential transmission chains. In drug resistance analysis, no PDR was detected in any large cluster, signifying that the current prevalent strain is still sensitive to first-line antiviral drugs. Existing treatment regimens can continue to be applied to this population.

The AIDS prevention and control situation in Chongqing was severe, requiring government leadership and multi-department collaboration. Commercial heterosexual contact remained a key transmission route for HIV, yet systematic research targeting this pathway remains insufficient. This study provides a basis for future research on the transmission dynamics in this population, and also offers empirical support for drug resistance monitoring and precise regional interventions.

## 5 Limitations

First, all sociodemographic information collected in this study relied on patient self-reports, potentially introducing recall bias and reporting biases. Second, as the study focused on cases infected through commercial heterosexual contact, the predominantly male sample composition means that the findings primarily reflect the transmission network characteristics of males infected individuals and cannot fully represent the transmission dynamics among FSWs infected through this route. Finally, due to resource constraints, comprehensive field investigations and interventions could not be conducted for the identified large transmission clusters. Subsequent research will be dedicated to addressing this deficiency. Moving forward, our team will expand molecular network surveillance coverage, monitor drug resistance dynamics, and optimize intervention strategies and measures based on these findings.

## 6 Conclusion

This study analyze the molecular transmission networks and drug resistance characteristics of newly reported individuals infected through commercial heterosexual contact in Yubei District from 2020 to 2024, revealing the HIV-1 epidemiological situation within this population. The HIV subtypes composition in this population is diverse, with CRF07_BC as the predominant genotype. CRF85_BC exhibiting high clustering. DRMs show low prevalence in patients with CHC. Enhanced surveillance and treatment management should target high-risk individuals aged ≥ 60 years, registered residents of Yubei District and infected with the CRF85_BC. Focused investigations and interventions are needed for identified large clusters.

## Data Availability

The gene sequences in this paper can be found in the repositories of “National Microbiology Data Center”. URL is https://nmdc.cn/resource/genomics/project/detail/NMDC10020686.

## Footnotes

1
https://comet.lih.lu

2
http://blast.ncbi.nlm.nih.gov

3
https://www.hiv.lanl.gov/

4
https://itol.embl.de/

5
https://hivdb.stanford.edu/
